# Evaluation of auditory processing and phonemic discrimination in children with normal and disordered phonological development

**DOI:** 10.1590/S1808-86942010000600015

**Published:** 2015-10-19

**Authors:** Tiago Mendonça Attoni, Victor Gandra Quintas, Helena Bolli Mota

**Affiliations:** 1 Master's degree in human communication disorders, Santa Maria Federal University. Speech therapist; 2 Master's degree in human communication disorders, Santa Maria Federal University. Speech therapist; 3 Doctoral degree in linguistics and languages, Pontifical Catholic University, Rio Grande do Sul. Associate professor at the Santa Maria Federal University. Santa Maria Federal University (Universidade Federal de Santa Maria)

**Keywords:** hearing, child, speech disorders

## Abstract

**Abstract:**

Auditory processing and phonemic discrimination are essential for communication. Type of study: Retrospective.

**Aim:**

To evaluate auditory processing and phonemic discrimination in children with normal and disordered phonological development.

**Material and Methods:**

An evaluation of 46 children was carried out: 22 had phonological disorders and 24 had normally developing speech. Diotic, monotic and dichotic tests were applied to assess auditory processing and a test to evaluate phonemic discrimination abilities.

**Design:**

Cross-sectional, contemporary.

**Results:**

The values of normally-developing children were within the normal range in all auditory processing tests; these children attained maximum phonemic discrimination test scores. Children with phonological disorders performed worse in the latter, and presented disordered auditory processing.

**Conclusion:**

Auditory processing and phonemic discrimination in children with phonological disorders are altered.

## INTRODUCTION

The myofunctional structures of the oral, auditory and central nervous system need to function normally for a child to acquire the sound of speech, otherwise disorders may become obstacles against such acquisition and the correct use of the phonological system.^1^, ^2^

At birth babies are able to detect contrasts in consonant sounds. They thereafter become able to selectively discriminate the phonemes of the language to which they are exposed. During this period, the perception of new sounds is reorganized and improved, which are essential for speech learning, which will only occur if the child is able to discriminate phonemes. These abilities improve with age because of experience and maturation.^3^, ^4^, ^5^

Some children - even without detectable etiological factors - encounter difficulties in using speech sounds correctly. Such speech difficulty is named phonological disorder, and is characterized by inadequate use of sounds (phoneme substitutions, omissions and distortions).^6^, ^7^

The severity of phonological disorder may be established by calculating the percentage of correct consonants (PCC),^8^ classifying such disorders as severe (PCC < 50%), moderate-severe (51% < PCC < 65%), moderate-medium (66% < PCC < 85%), and medium disorder (86% < PCC < 100%). This proposal for measuring the severity of speech disorders is based on gathering and analyzing speech samples.

A few authors,^9^, ^10^ by analyzing auditory discrimination in children with phonological disorder, have suggested that these children have phonemic discrimination difficulties. This ability makes it possible for a person to differentiate two closely similar speech sounds.

Additional methods are behavioral tests to assess auditory processing; these are used to evaluate phonological disorders.^11^, ^12^, ^13^ The hearing system takes auditory input to support efficient reading, by mechanisms and processes of auditory discrimination such as: location and lateralization of sound; recognition of auditory patterns; temporal aspects of hearing (temporal integration, temporal discrimination, temporal order and marking); and perception of competitive and low-redundancy sounds. Disorders of auditory processing are disorders in any of these abilities.^14^

Based on these ideas, the purpose of this study was to study the performance of children with normal and with altered phonological development, looking at tasks involving auditory processing and discrimination of phonemes; a possible association between the two was also sought.

Studying specific populations with communicative representation difficulties may be helpful to understand speech processes. This justifies the need for further studies on how children with phonological disorders deal with auditory stimuli, which is apparently an essential ability to acquire speech sounds.

## MATERIAL AND METHOD

This study included 46 male and female children aged from 5 to 7 years, of which 22 had a diagnosis of phonological disorder (study group) and 24 had normal speech development (control group). Children with phonological disorders were selected in screening visits to the speech unit of the Phonology Service of the Santa Maria Federal University in Santa Maria, RS. Normal speech children were selected from children of a philanthropic school in the same city; parents were asked to allow their children to participate voluntarily. Explanations about the purposes and procedures of this study were given, after which evaluations were carried out if parents had authorized their children to participate.

The institutional review board approved this study (protocol no. 23081.006440/2009-60); parents or caretakers signed a free informed consent form for children to participate.

The evaluations of both groups of children took place at a school clinic of a higher education institution, and were carried out by the main researcher.

All children underwent a phonological assessment to investigate the stomatognathic system, language, to carry out an ontological inspection and audiological testing (pure tone audiometry, speech reception threshold, and the speech perception and recognition rate), and to study the acoustic reflex, the tympanometric curve, and speech analysis.

Assessment of phonetic skills in children^15^ was applied to study the phonological system; it consists of obtaining and analyzing a sample of speech. This instrument gathers spontaneous speech to name and detect the production of Brazilian phonemes.

The simplified auditory processing evaluation, the pediatric speech intelligibility (PSI) test, the speech-innoise test, the staggered spondaic word (SSW) test, and the dichotic digit test were applied to assess auditory processing.^16^

Participating children were subdivided and organized as follows:

a control group comprising 24 children (8 male and 16 female) with normal speech development.

a study group comprising 22 children (12 male and 10 female) with phonological disorders.

Inclusion criteria for the control group were:

1) no phonological disorders;
2) no apparent neurological, emotional or perception disorders, anatomical or physiological alterations in phonoarticulatory organs, or altered language expression and understanding;
3) normal hearing;
4) being right-handed;
5) no complaint of altered auditory processing.

Exclusion criteria for the control group were communication disorders. These children were required to perform well in speech, language, and phonoarticulatory and auditory organ tests.

Inclusion criteria for the study group:

1) having a phonological disorder;
2) no apparent neurological, emotional or perception disorders, anatomical or physiological alterations in phonoarticulatory organs, or altered language expression and understanding;
3) normal hearing;
4) being right-handed;
5) no past or current phonoaudiological therapy.

Exclusion criteria for this group were any perceivable communication disorders. A phonological disorder should be present in its purest form, without apparent etiologies.

A Fonix FA - 12 clinical audiometer with TDH 39 earphones (ANSI S3.6/96: ANSI S343/92 calibrated) was used. Stimuli were given in an acoustic booth. An AZ& impedance meter with TDH 39 earphones and 220 Hz at 70 dB probes was used for investigating contralateral acoustic reflexes at 500 to 4,000 Hz (ANSI S3.6/96: ANSI S343/92 calibration). Volume 1 and volume 2 CDs with test recordings and posters illustrating the answers for the PSI test were used, according to the manufacturer's instructions.^16^

Auditory processing was assessed with diotic, monotic and dichotic listening tests.

### Diotic listening

Agogos, coconut shells, jingle bells and bells were used in the simplified auditory processing evaluation. This test requires no sophisticated mechanisms; it is easily applied. It consists of three steps: localization of sound - the child needs to find the direction of the sound stimulus, and to do it correctly 4 out of 5 times; errors are expected to be in front of or above the head. The next step is verbal sequence memory: syllables are presented and children are expected to repeat at least two of three presented sequences. Next, we have nonverbal sequence memory: children here need to listen to sounds of predetermined instruments (jingle bells, agogo, bells, coconut shells) and to correctly recall the sequence in two of three presented sequences.

### Monotic listening

The PSI with ipsilateral competing messages (PSI/ICM) is a sentence-recognition by pictures test. The competing message is a story the child needs to ignore. Attention should focus on the commando to indicate the corresponding figure. The auditory abilities evaluated here are figure-background and audiovisual association.

The speech-in-noise test evaluates message degradation or reduction because of competing noise. Children were told that they would hear a series of words and noise, and they were to repeat the words; this test evaluates the figure-background ability.

### Dichotic listening

The SSW test consists of presenting 40 items comprising four paroxytone disyllables totaling 160 words. Two words are presented simultaneously to opposite ears; the second syllable of the second word overlaps the first syllable of the third word. There are thus four conditions for stimuli: DNC (direct non-competing), DC (direct competing), LC (left competing), and LNC (left non-competing). We adopted the suggestion in the manual,^16^ analyzing only the competing conditions. Children are asked to repeat all words in the order they were presented. Abilities analyzed here are auditory analysis and synthesis, temporal ordering, and memory.

The dichotic digit test (free and directed attention step) consist of presenting four times a lists of disyllable digits in Brazilian Portuguese; four different digits are presented simultaneously, two to each ear, thereby characterizing a dichotic test. This test identifies selective attention abilities (directing listening).

The phoneme discrimination test with figures was applied. This tool was developed to assess phoneme discrimination in children aged 4 to 8 years. The phoneme discrimination test with figures presents 60 words comprising 30 minimum pairs (words with the same syllabic structure differing by only one phoneme) organized into 40 presentations. Each presentation contains three cards, each containing two drawings. Children listen to two auditory stimuli (two words) and point to the corresponding figure.^17^

Statistical treatment was based on the SAS software user's guide: Statistical Analysis System Institute, Inc., Cary, NC, 2001; the Pearson coefficient correlation test.

## RESULTS

The PSI mode test (PSI/ICM) was carried out successfully in the control and study groups. There was no margin of error in both groups, that is, all achieved 100% correct answers. There was also no time or other differences to characterize the groups in relation to each other.

The results of the speech-in-noise test were similar to those of the PSI test. The same applied to the phoneme discrimination test with figures results in the control group.

Thus, there were no true possibilities of carrying out statistical analyses, since there were no differences or errors to be analyzed.

The data presented below are those with statistical significance.

The quantitative and qualitative results of the SSW test in the control group remained within normal limits.

The quantitative data were below the expected normal results in the study group; there were many omissions, switches and type A response patterns in the two conditions (right competitive and left competitive). Furthermore, children required more time to answer.

Table 1 shows the quantitative values of the control and study groups.

**Table 1:** Result of the SSW test in the control and study groups based on the number of errors

Variables	Control group	Mean	SD	Minimum	Maximum

RC	24	7.68	5.13	0	17.0
LC	24	8.68	6.46	0	15.0

Variables	Study group	mean	SD	Minimum	Maximum

RC	22	65.50	10.39	40.0	75.0
LC	22	58.50	10.01	40.0	75.0


RC= right competitive; LC= left competitive; SD= standard deviation

Table 2 shows the dichotic digit test results, showing the number of errors in each group, the means, standard deviation, and minimum and maximum levels. In the free attention steps, the response rates (right and left ears) did not differ statistically in both groups. Abnormal values were found in the study group.

**Table 2:** Results of the dichotic digit test in number of errors in the control and study groups

Variables	Control group	Mean	SD	Minimum	Maximum

FA	24	4.33	2.47	2.0	8.0
ARE	24	1.66	3.44	0	9.0
ALE	24	0.16	0.81	0	4.0

Variables	Study group	Mean	SD	Minimum	Maximum

FA	22	39.22	8.70	20.0	56.0
ARE	22	20.27	10.12	2.0	34.0
ALE	22	19.77	8.10	0	36.0

FA= free attention; ARE= attention in the right ear; ALE= attention in the left ear; SD= standard deviation.

Only the results of the study groups were altered in the simplified auditory processing evaluation. Table 3 shows the performance data of children in the control and study groups.

**Table 3:** Distribution of the simplified auditory processing evaluation values.

Variables	Control group	Mean	SD	Minimum	Maximum

IS	24	2.91	0.28	2.0	3.0
VS	24	5.29	0.46	5.0	6.0
SLT	24	4.91	0.40	4.0	6.0

Variables	Study group	Mean	D P	Minimum	Maximum

IS	22	1.81	1.00	0	3.0
VS	22	4.90	1.19	3.0	6.0
SLT	22	4.04	0.95	2.0	5.0

SI= instrument sounds; SV= verbal sounds; TLS= sound localization test; SD= standard deviation.

The phoneme discrimination test with figures values in the control and study groups are represented on Table 4; it includes the mean, standard deviation, and minimum and maximum values.

**Table 4:** Result of the phoneme discrimination figure test

Variables	N	Mean	SD	Minimum	Maximum

Control group	24	40.0	0.00	40.0	40.0
Study group	22	34.0	0.78	34.0	37.0

SD = standard deviation; N= subjects.

## DISCUSSION

Two parallel neurobiological pathways have been implied directly in auditory processing. One for discriminating speech-related auditory stimuli, and the other on how subjects deal with linguistic abilities - the phonological system.^18^, ^19^

This study is based on these precepts; if there is a connection between these interfaces, disorders in one pathway can affect the other.

The results show that the control group had maximal results in the phoneme discrimination test with figures, PSI, speech-in-noise tests, normal SSW test, dichotic digit test and the simplified auditory processing evaluation. This did not apply to the study group, in which altered results were found in the dichotic listening tests, and there were error patterns in the phoneme discrimination test with figures. Auditory processing disorders lead to altered perception for tasks involving discrimination of phonemes and use of speech sounds.

Both groups reached similar results in the speech-in-noise and the PSI tests. All children scored the maximum values. We concluded, based on our analysis of the auditory abilities involved in this task, that children with phonological disorders do not find it difficult to code auditory messages (loss of selective attention; expressive language; understanding difficulties; distraction; behavioral issues; dysgraphism). They were able to assimilate messages when these were given in degraded form, to separate different acoustic signals, to focus on attention, and to create audiovisual associations.^20^ The results of these two tests were equal among all children. A study^21^ that applied the PSI and speech-in-noise tests (monotic mode) in children with learning difficulties revealed significant correlations between the percentage of correct answers and increased age.

Data quantification in the dichotic listening test results (SSW test) was altered in the study group; there was also a high rate of errors in the qualitative section of the test, suggesting that children with phonological disorders encounter difficulties in auditory analysis and synthesis, temporal ordering, and memory. These abilities appear to be relevant for phonological acquisition and phoneme-grapheme associations.^22^ These findings concur with those of other studies that applied the SSW test to study these abilities in children.^12^, ^23^

The dichotic digit test results in the study group were highly compromised. The children generally were unable to separate the acoustic signal by using selective attention. On the other hand, there was a small advantage in the number of correct answers in the SSW test. Answers in digits appear to be better understood and memorized when compared to stimuli by sentences and syllabic structures. An explanation may be the memory component of work - the central executive function - that focuses on the acquisition of vocabulary, and information processing, storage and evocation.^24^ Digits have an advantage over words because the semantic load is lower.

The simplified auditory processing evaluation results were also altered in the study group; there was a significant directly proportional relation in errors between verbal and non-verbal sounds. The ability to memorize sequences was compromised, especially compared to the control group. Similar results have been published of school-aged children with phonological disorders.^16^

It is possible to see difficulties in the children enrolled in this study by analyzing the two dichotic tests and the dichotic listening test. The type of auditory processing disorder may be defined by poor decoding ability (impaired writing; phoneme analysis and synthesis difficulties; integrating the acoustic aspects of speech), and organization of auditory information (organizing acoustic events in time and ordering speech sounds),^16^ which underline the connections between phonoarticulatory disorders and altered auditory processing. Disordered auditory abilities make it impossible to correctly use speech phonemes.^25^

The phoneme discrimination test with figures is a proposed evaluation; it cannot measure any value to define whether children have phonemic discrimination difficulties. The control group data guided us in measuring the effect of errors. All participants in the control group attained the maximum scores. There were errors among participants of the study group, although the scores were not low. Tasks are apparently simple, but require paying attention, discriminating the stimuli, making lexical and audiovisual associations. These surveys occur more naturally and effortlessly among adults; for normal children, however, these tasks have low redundancy (the acoustic cues are less easily perceived).^26^ The perception appears to decrease in children with phonological disorders.

It should be noted that the mean [+/-voice] scores were among the lowest. The sonority of differentiated word pairs in this criterion is different; the voiceless phonemes are produced without vocal fold movements and the voiced phonemes are produced with vocal fold movements. The main method for differentiating voiceless from voiced sounds is to use the time elapsed between the release of the stop phoneme and the beginning of voicing - the voice onset time (VOT).^27^ Results were statistically significant in a study assessing auditory discrimination and its repercussion on speech and writing abilities; this became clear when evaluating VOT function.^28^

The results of auditory processing and phoneme discrimination tests were positive and almost error-free in the control group. On the other hand, each evaluation contained errors in the study group. Thus, there is a relation between auditory processing abilities and phoneme discrimination, and their effect on speech sound assimilation and use. An inability to decode and organize auditory stimuli is associated with speech difficulties, which in turn results in decreased phoneme discrimination abilities in children with phonological disorders. It is interesting to note that the anatomical sites and physiological pathways involved in these processes are basically the same.^29^ The data concurs with that of another study which investigated these correlations in pre-school children at a high risk for dyslexia.^30^

Logically speaking, phonological acquisition-related process and discrimination disorders may compromise the incorporation and reorganization of speech sounds.

Thus, evaluations of auditory processing and phonemic discrimination should be carried out jointly in cases of speech disorders for a more certain diagnosis and effective therapy.

## CONCLUSION

Children with phonological disorders present difficulties in auditory processing and discrimination of phonemes.

Auditory processing disorders affect the ability to discriminate phonemes; the signs of these abnormalities may arise when using speech sounds.
